# Dependability enhancing mechanisms for integrated clinical environments

**DOI:** 10.1007/s11227-017-2003-0

**Published:** 2017-03-29

**Authors:** Wenbing Zhao, Mary Q. Yang

**Affiliations:** 1 Department of Electrical Engineering and Computer Science, Cleveland State University, Cleveland, OH 44115 USA; 2 Department of Information Science, George Washington Donaghey College of Engineering and Information Technology, Little Rock, AR USA; 3 Joint Bioinformatics Program of University of Arkansas at Little Rock, University of Arkansas for Medical Sciences, 2801 S. University Avenue, Little Rock, AR 72204 USA

**Keywords:** Integrated clinical environments, Cyber security, Service integrity, Continuous availability, State machine replication, Byzantine agreement

## Abstract

In this article, we present a set of lightweight mechanisms to enhance the dependability of a safety-critical real-time distributed system referred to as an integrated clinical environment (ICE). In an ICE, medical devices are interconnected and work together with the help of a supervisory computer system to enhance patient safety during clinical operations. Inevitably, there are strong dependability requirements on the ICE. We introduce a set of mechanisms that essentially make the supervisor component a trusted computing base, which can withstand common hardware failures and malicious attacks. The mechanisms rely on the replication of the supervisor component and employ only one input-exchange phase into the critical path of the operation of the ICE. Our analysis shows that the runtime latency overhead is much lower than that of traditional approaches.

## Introduction

Information and communication technology has been penetrating to many fields to enable automated operations, which not only improves efficiency and productivity, but also increases safety. In recent years, it has been recognized that medical devices could be interconnected together to streamline the workflow of clinical operations and significantly improves the safety of patients. This effort has led to the specification of integrated clinical environment (ICE), which aims to standardize how medical devices should interoperate together and work with decision-making systems [1]. One of the main functionalities of an ICE is to ensure automated safety interlocks of operations, which is prone to human errors [15]. For example, during a major surgery, a ventilator may be used to provide the patient with oxygen and laser may be used for precise incision. When the surgeon is ready to use the laser, the ventilator flow must be temporarily turned down or off, and it must be turned back up to the normal level once the surgeon finishes using the laser. If someone forgets to turn down the ventilator flow when the laser is turned off, a fire hazard could ensue. On the other hand, the consequence of failing to turn the ventilator back up after the laser operation could cause the death of the patient.

**Fig. 1:**
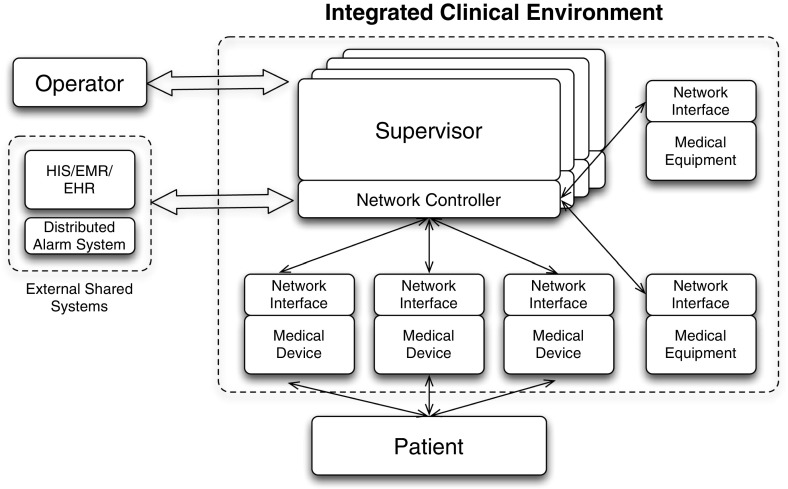
Main components in an ICE

An ICE interconnects medical devices and equipment for automated patient care before, during, and after a clinical operation of a patient [1]. The main components in an ICE are illustrated in Fig. 1. Most of these components are self-explanatory. The difference between the medical device and the medical equipment is that a medical device is connected directly to the patient while the equipment is not. The supervisor is the most important component in an ICE because it takes the input from medical devices and equipment, consults with medical records of the patient via an external health information system, and coordinates all commands and controls of the system, including ensuring safety interlocks and alarm generations. Because the supervisor must communicate with other entities in the system via the network controller, we include the network controller as part of the supervisor.

The purpose of ICE dictates that it operates as a safety-critical real-time distributed system. An ICE operates in rounds according to a predefined time period. In each round, devices report to the supervisor regarding their status, and based on the input, the supervisor would compute the commands for the round. Considering the critical role played by the supervisor component, it must be hardened to be resilient to both hardware failures and malicious attacks. Even though dependable computing has been studied for several decades, most solutions are designed for general-purpose distributed systems without real-time and safety restrictions [4, 27]. How to enhance the dependability of safety-critical real-time systems is challenging, and it has rarely been studied in the past, especially in the presence of malicious attacks.

In this article, we propose a set of lightweight mechanisms to protect the supervisor component from both partial hardware failures and malicious attacks, which will enhance the dependability and trustworthiness of ICEs. First, the supervisor is replicated. Space redundancy is necessary to protect the supervisor from partial hardware failures while offering continuous high availability, which is essential for ICEs. Second, by exploiting the fact that the supervisor logic is stateless (*i.e., * it does not keep temporary state on behalf individual clients and its output is completed derived based on the inputs it has collected), we are able to protect the integrity of the supervisor operation without resorting to the use of traditional Byzantine fault tolerance algorithms. We introduce only one input-exchange phase, which in the optimal case causes the delay of a single communication step along the critical path of ICE operations. Furthermore, our mechanisms do not require any replica to serve as the primary and all replicas are peers of each other. Hence, our mechanisms avoid having to deal with the issue of leader elections (referred to as view changes) when the primary replica fails, which is highly expensive and will cause unpredictability to the operation of ICEs.

Our mechanisms protect the integrity of ICEs from malicious supervisor replicas and ensure that (1) commands issued by a faulty supervisor replica will not be accepted by any nonfaulty medical devices (unless it behaves as a nonfaulty replica and generates the same commands as those of a nonfaulty replica), and (2) no two nonfaulty supervisor replicas issue different commands for the same round (it is acceptable that some nonfaulty replicas are not able to issue any command).

This article is organized as follows. Section 2 discusses related work. Section 3 specifies our system model. Section 4 describes the lightweight mechanisms to enhance the dependability of ICEs. Section 5 concludes this article.

## Related work

The possibility of using closed-loop control to ensure patient safety in an ICE was studied in [3, 16]. In this study, a specific scenario was considered where the supervisor would collect information from a pulse oximeter. If the information shows that the patient has shown signs of pain medicine overdose, the supervisor would ask the PCA pump to stop releasing more pain medicine. The study demonstrated that closed-loop control is achievable in an ICE. However, it pointed out that the network must be reliable and guarantee tightly bounded message delivery.

In [13], Kim et al. introduced a network-aware supervisory system (NASS) for ICE. The system has two layers. The first layer allows the implementation of the supervisory logic using a synchronous network model. The second layer adds safety provisions by handling network failures. The system operates in rounds. In each round, the supervisor collects information from each device and computes a set of commands for each device for the next round. An interesting contribution is distribution of a command vector to each device instead of a single mode. If a device fails to receive a new command vector, it would execute the next mode in the vector. When it reaches the last mode in the vector, it switches to the fail-safe mode.

In this research, we follow and extend the open-loop design proposed in [13]. This open-loop design imposes a strong assumption that the supervisor is trusted [13]. We extend this design by using a set of lightweight mechanisms to protect the supervisor component from hardware failures and cyber attacks.

Later, Kang and Wu et al. [12, 21] proposed an organ-based hierarchical coordination architecture for ICE. In this architecture, several levels of controllers are used so that they can make localized control decisions for medical devices at the same time. The advantage of this proposed hierarchical architecture is that the system is more resilient to network failures because the network failure of one section might not impact other sections. However, allowing concurrent and localized decisions inevitably raises the global consistency issue. If not handled properly, the inconsistency could lead safety violations. To achieve a global consistency, a lock-based coordinator protocol is employed. This additional mechanism adds complexity of the system. In the presence of malicious attacks, this additional protocol must also be protected. Hence, we favor the flat architecture for the reduced complexity and robustness.

In addition to safety issues, the security of ICEs has also been studied. In [11], the secure communication requirements for ICE were elaborated and the applicability of existing security protocols in ICEs was discussed. Furthermore, common attacks on ICE and possible mitigation methods of these attacks were introduced in [19, 20]. In a later work [18], the threats on a particular ICE were analyzed in the context of patient pain medication management. However, similar to previous research, the supervisor was assumed to be trusted. Attention was placed on the attacks by compromised devices or malformed messages over the network. We disagree with this important assumption for two reason. First, medical devices are directly attached to the patient. Actuating devices, such as PCA pumps, can put the patient in grave danger directly, if they are compromised. It does not seem logical to talk about the attacks from these devices. Second, an external adversary cannot easily access medical devices because they must go through the supervisor first before reaching to the medical devices according to the ICE architecture. It is inconceivable for an adversary to penetrate a medical device without compromising the supervisor first. Strong physical security in operating rooms would help protect medical devices from being attacked. That is why in this research, we focus on the protection of the supervisor.

This research is also related to Byzantine fault tolerance. Byzantine fault tolerance has been intensely studied since Castro and Liskov revitalized this research field [4, 25]. The strategy employed in this research is along the line of application-aware Byzantine fault tolerance [5–7, 23, 24, 28–30]. The essence of this strategy is to minimize of use of traditional Byzantine agreement algorithms, which typically incurs high runtime overhead (in terms both latency and throughput), by exploiting application semantics [27]. In this research, we are able to avoid using full Byzantine agreement algorithm because the supervisor operation is stateless. Previously, we investigated the same issue in a conference publication [26]. However, a full Byzantine agreement is used for every round of operation, which not only adds latency, but adds uncertainty to the system as well due to possible view changes caused by the failures of the primary replica. As we will show in Sect. 4 of this article, our lightweight mechanisms incur about half of the latency overhead of what we proposed in [26] when the primary replica is not faulty. When the primary replica is faulty and one round of view change is needed, the latency overhead for the traditional approach we proposed in [26] is significantly higher than that of the lightweight mechanisms, and it increases nonlinearly with respect to the replication degree.

## System model

Similar to [13], we assume that medical devices normally operate correctly. When a device suffers from a hardware failure, it would switch to a predefined fail-safe mode for patient safety. Furthermore, we assume that there are no malicious insider adversaries because they would be free to bypass strong physical security in operating rooms and arbitrarily manipulate medical devices and equipment, which would make it impossible to defend using any computational algorithms and mechanisms.

We assume that the supervisor is replicated for fault tolerance. $3f+1$ supervisor replicas are needed to tolerate up to *f* faulty replicas. Each replica has a monotonically increasing replica id *k* (from 0 to 3*f*). We assume a bounded processing delay at all components. Furthermore, we assume that message transmission, propagation, and delivery are also bounded *normally*, but we do allow the network to exceed the bound occasionally.

It is intuitive to assume or require that a new round of commands can be sent to all medical devices atomically. Unfortunately, this assumption would make the system very vulnerable to disruptions in the network. If the commands for the current round are not delivered atomically, one actuating medical device may execute the new command while another act on the next command from the command vector received previously. This could result in safety violations. Consider the airway-laser surgery as an example. The laser must not activate until the oxygen flow has been reduced by the ventilator. If one assumes that the command to turn on the laser and the command to block the oxygen flow at the ventilator can be delivered atomically, and the two commands are issued in the same round, nonatomic delivery of the two commands will lead to a serious safety problem. The mechanism introduced in [13] helps to some extent by suppressing the new command vector when one or more devices fail to acknowledge the receipt of the command of the previous round. However, the mechanism still could not address the fundamental atomic command delivery issue because it cannot guarantee that the new command vector can reach all medical devices in the current round. If a device receives the new command, it would execute the new command. On the other hand, if another device fails to receive the new command in time, it would have to execute the next command from the previous command vector. Unless the new command vector and the previous command vector are carefully crafted, the safety risk might still occur.

In our opinion, ensuring time-bounded atomic command delivery to multiple devices across the network while the network cannot guarantee absolute real-time bound is an intractable problem. Hence, instead of pursuing atomic command delivery, a more reasonable approach is to remove the need for atomic command delivery by spacing inter-locking commands in different rounds. Using the same airway-laser surgery example, instead of instructing the laser to turn on and the ventilator to block the oxygen flow in the *same* round, the supervisor logic should ask the ventilator to block the oxygen flow in one or two rounds before asking the laser to turn on. For the reverse operation, we want to make sure that the laser has already been turned off (in a previous round) before the supervisor logic issues a new command to turn on the oxygen flow at the ventilator. By sending inter-locking commands in different rounds, we can rely on the acknowledgment of the previous round to decide whether or not to issue a new command to turn on the laser. Hence, we could remove the atomic command delivery requirement while ensuring the safety of ICEs.

Because an ICE operates in rounds and a new set of commands is computed based on the input provided by the medical devices and equipment, the supervisor is stateless. This gives us the opportunity to significantly reduce the complexity of the mechanisms needed to enhance the dependability of ICEs.

## Correctness properties

Our mechanisms are designed to satisfy three correctness properties. The three correctness properties specify the consistency, liveness, and safety requirements for the ICE.

- Consistency property: All nonfaulty medical devices accept the same set of commands in each round.
- Liveness property: A faulty supervisor replica cannot prevent an ICE from making progress when a progress should be made according to the system condition.
- Safety property: Patient safety is guaranteed as long as no more than *f* supervisor replicas fail.

It is well known that it is impossible for processes in a distributed system to reach a consensus if the system is asynchronous and processes in the system might be subject to crash faults [10]. In order to ensure processes in such a system to reach a consensus, compromises are typically made, such as the use of an unreliable failure detector [8]. Our mechanisms do not require atomic command delivery even though it is quite intuitive to require this for consistency. By implementing the inter-locking mechanism in two or more rounds instead of the same round, we relax the requirement on the system and on the network. By doing this, we are imposing a hard time bound on the operation of the system. If no message is received within the maximum round period, we claim that the message is lost and proceed forward based on this assumption. Essentially, we are using a form of unreliable failure detector to work around the impossibility result. Hence, we can ensure the consistency property with our mechanisms.

## Mechanisms for enhancing the dependability of ICE

In this section, we first describe a set of mechanisms for enhancing the dependability of an ICE and then provide a proof of correctness of the mechanisms.

### Dependability mechanisms

**Fig. 2:**
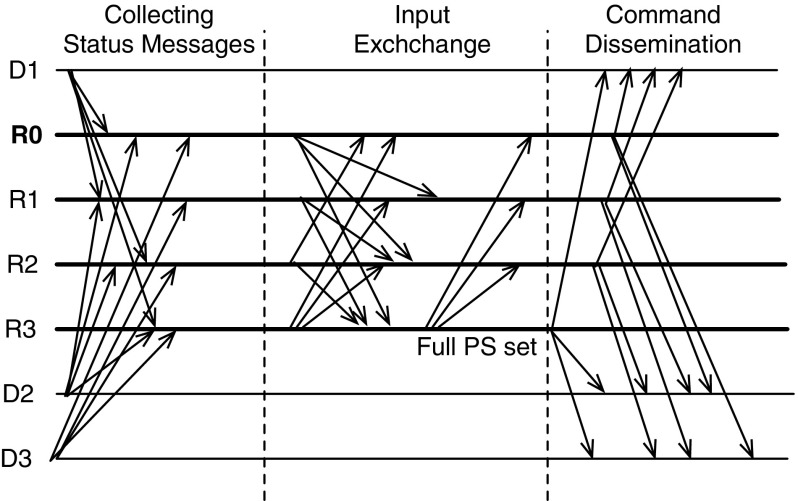
The main phases in one round of ICE operation. *R*0, *R*1, *R*2, *R*3 denote the supervisor replicas, and *D*1, *D*2, *D*3 denote medical devices. Here we assume that *R*3 is able to collect the entire PS set from all devices, in which case, *R*3 sends the set to all other replicas

As shown in Fig. 2, we add an input-exchange phase between the existing input phase and the command dissemination phase in each round of operation of an ICE.

#### Input phase

In this phase, all connected medical devices report their status to all supervisor replicas. The status message has the form of $\langle$ status $,r,t,d,P\rangle _{\sigma _d}$, where *d* is the device identifier, *r* is a monotonically increasing round number, *t* is the timestamp of the status message (to prevent replay attacks), *P* contains the device state and the acknowledgment for the last command received, and ${\sigma _d}$ is the digital signature signed by device *d*. A device must use a one-to-many reliable multicast mechanism to ensure that its message will reach all nonfaulty supervisor replicas.

#### Input-exchange phase

In this phase, a replica first keeps collecting the status messages sent by different devices. After the replica has collected messages from all devices, or a predefined timeout has occurred, it multicasts the set of status messages that it has collected to other replicas. This phase increases the likelihood of all nonfaulty replicas receive all status messages within the current round. The input-exchange message has the form $\langle$ input-exchange $,r,i,\textit{PS} \rangle _{\sigma _i}$, where *i* is the replica identifier, and *PS* is the set of original status messages signed by the medical devices.

A replica keeps collecting the input-exchange message sent by other replicas until either it has received from all other replicas, or a predefined timeout has occurred. This timeout is set so that it is guaranteed that the replica can receive at least from 2*f* other replicas. Subsequently, each replica would examine the collected *PS* set to build a superset of the status for hopefully all devices. The status for a device is included in the superset if it is in any of the *PS* set.

To further increase the probability for more replicas to receive the status messages sent by all medical devices, a replica multicasts the complete *PS* set to all other replicas if it could build a complete set of status messages (one from every medical device).

#### Command dissemination phase

If and only if a replica manages to receive the status message from every medical device, it computes the commands for the next round of operation and subsequently multicasts them to all medical devices. The message takes the form $\langle$ command $,r,t, i,\textit{PS},O \rangle _{\sigma _i}$, where *t* is the timestamp of the message, *i* is the identifier of the sending replica, *PS* is the complete set of signed inputs from all medical devices, and *O* is the set of command vectors computed by replica *i*. If a replica could not receive input from all medical devices, it won’t generate a new command because the supervisor might not be able to compute the next set of commands if it is missing information regarding the status of the medical devices.

On receiving a command vector from a supervisor replica, a device verifies the digital signature for the command, makes sure that the round number *r* is indeed the current round, and verifies every digitally signed status message sent by medical devices. If the message passes all these tests, the device buffers the message prior to executing it. A medical device will not accept the new command vector and execute the first one in the vector until it has collected matching command vectors from at least $f+1$ different supervisor replicas. By matching, we mean that two messages have identical *r*, *t*, *i*, *S*, *O* components.

### Proof of correctness

In this section, we prove that our mechanisms satisfy the consistency, liveness, and safety correctness properties.

#### Consistency property

*All nonfaulty medical devices accept the same set of commands in each round.*

##### Proof

Because we assume that all medical devices are semi-trusted, they all attempt supply their status honestly to all replicas of the supervisor. Due to network delays, some of the status messages might not reach some or all supervisor replicas. Furthermore, a replica generates a new set of commands only if it has received the status message from every medical device (directly or indirectly). Because no supervisor replica could possibly fabricate any status message, all nonfaulty replicas must have computed the new set of commands based on the same set of status messages, which would lead to the same set of commands. Furthermore, because a medical device must have collected $f+1$ matching command sets, and there are at most *f* replicas can be faulty, at least one of the $f+1$ matching command sets is generated by a nonfaulty replica. This proves the consistency property. $\square$

#### Liveness property

*A faulty supervisor replica cannot prevent an ICE from making progress when a progress should be made according to the system condition.*

##### Proof

By progress, we mean that all devices could receive a new set of commands and execute them in the current round. This will happen when all medical devices have successfully reached all nonfaulty supervisor replicas in the current round (directly or indirectly). The faulty replicas (at most *f* of them) could collude and attempt to stall the progress by not forwarding the status messages received to other replicas. However, because there are other $2f+1$ or more nonfaulty replicas, they can work together to propagate the status messages they have received to increase the likelihood that other replicas could receive them as well by forwarding such messages to each other.

Faulty replicas could also collude together by not sending any commands to the devices. This won’t be successful either because there are $2f+1$ or more other nonfaulty replicas that would send their commands to all devices. $\square$

#### Safety property

*Patient safety is guaranteed as long as no more than* *f* *supervisor replicas fail.*

##### Proof

In an ICE, a safety violation is caused by incompatible actions taken by different medical devices. When supervisor is replicated, our mechanisms guarantees that if two or more nonfaulty replicas issue the command vectors for the current round, they must be consistent. It is possible some nonfaulty replicas won’t be able to issue a new command vector because of the lack of status messages. However, this will not cause different medical devices to accept conflicting command vectors. Therefore, the safety property holds. $\square$

## Cost analysis

In this section, we analyze the latency overhead of our mechanisms. Our mechanisms only introduce a single additional phase, the input-exchange phase, in each round. We use $D_n$ to represent the worst-case latency for transmission and propagation of a message, and $D_p$ to represent the worst-case latency for each execution step at each supervisor replica. By execution step, we mean an action taken at a replica, such as digitally signing a message, verifying the digital signature of a message, and logging of messages. Because messaging signing and verification are significantly more expensive than other local processing, we only consider these two execution steps and ignore other local processing.

During the input-exchange phase, a replica signs an input-exchange message and transmits the message, and then it verifies and collects up to 3*f* such messages sent by other replicas. Note that when verifying a message, a replica must not only verify the digital signature of the input-exchange message itself, but each status message in the *PS* set. For the analysis, we assume there are *n* number of medical devices. To optimize the verification process, a replica performs a full digital signature verification for each medical device only once (*i.e., * the first time the replica receives the status message from a device). If a status message is valid, the replica produces a secure hash of the message and stores it in its data structure. For repeated status message received, the replica simply compares the stored hash and the hash of the later one. Hashing a message and comparing two hashes are very fast operations. Hence, we omit such cost from our analysis.

Furthermore, a replica might have to wait until it receives a rebroadcast message sent by another replica that has received the status messages from all medical devices. Hence, in the worst case, the total delay in this phase, $D_{ix}$, is the sum of the following delays: (1) two communication steps (*i.e.,* $2D_n$), one for the input-exchange message, and the other for the rebroadcast; (2) digitally signing the input-exchange message at the replica (*i.e.,* $D_p$); (3) digitally signing the rebroadcast message at another replica (*i.e.,* $D_p$); (4) $3f+1$ actions on verifying the digital signature of the 3*f* input-exchange messages and the one rebroadcast message [*i.e.,* $(3f+1)D_p$]; (5) *n* number of actions on verifying the digital signature of the status message sent by *n* medical devices. Hence, $D_{ix}$ is defined by Eq. 1:

1 $$\begin{aligned} D_{ix} = 2D_n + 2D_p +(3f+1)D_p + n D_p = (3f+3+n)D_p + 2D_n \end{aligned}$$

Note that the above worst-case analysis holds regardless whether or not there are faulty supervisor replicas. This is very different from the case when a traditional Byzantine agreement algorithm is used, which requires the use of a primary replica. The use of special replica makes the algorithm subject to additional attacks on the primary replica, which could lead to one or more rounds of view changes (*i.e., * leader elections). View change algorithms are notoriously costly and unpredictable.

**Fig. 3:**
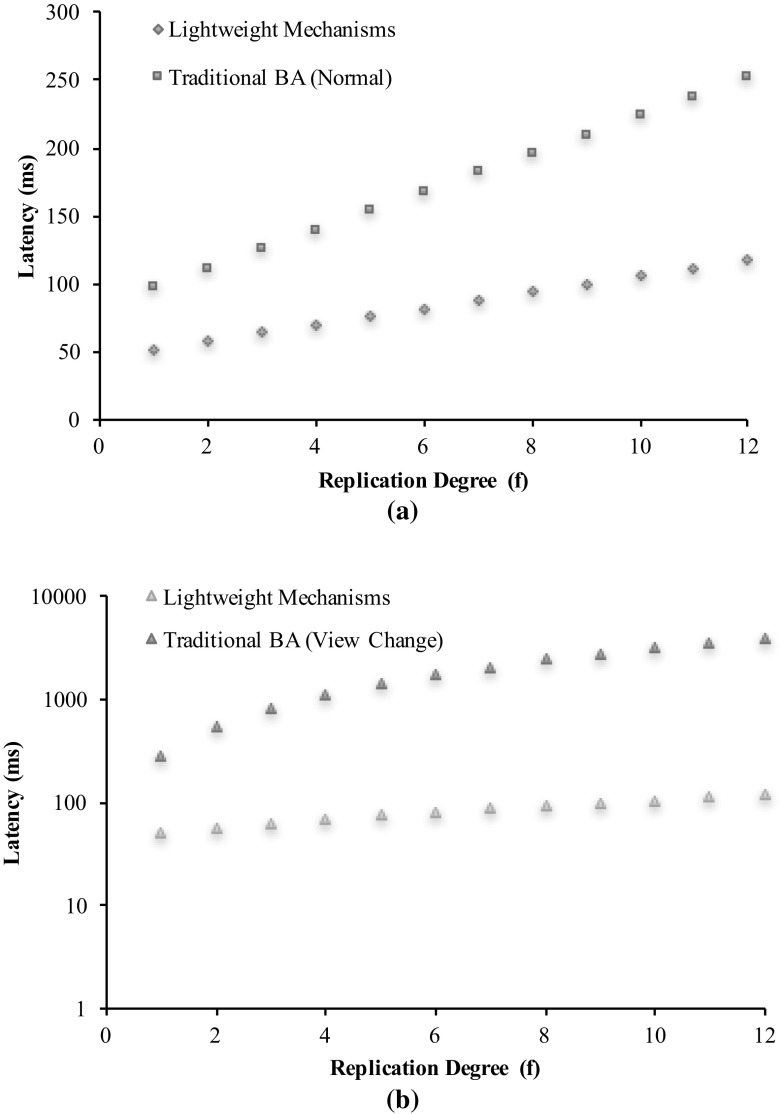
The latency overhead for our lightweight mechanisms in comparison with the traditional approach where a Byzantine agreement is used. **a** The latency when the primary replica is not faulty. **b** The latency when the primary replica is faulty and a view change is involved

According to Eq. 1, the latency overhead we introduce is linearly proportional to the following parameters: (1) replication degree *f*; (2) the worst-case processing delay $D_p$; (3) the worst-case message delivery delay $D_n$; (4) and the number of medical devices. The relationship between the latency overhead and the replication degrees *f* is shown in Fig. 3 (using the parameters $D_p=2\,\mathrm{{ms}}$, $D_n=10\,\mathrm{{ms}}$, and $n=10$). As shown in Fig. 3a (the curve labeled as Lightweight Mechanisms), the overhead is lower than $120\,\mathrm{{ms}}$ even when $f=12$. This is significantly below the 200*ms* round period given in [13]. Hence, the latency overhead introduced by our mechanisms is insignificant and would allow the ICE to operate normally.

To demonstrate the advantages of using our lightweight solution, we also include the comparison with our previous work [26], which used a full round of Byzantine agreement to ensure the consistency of the commands generated by the supervisor replicas. If the primary replica is not faulty, the consensus can be reached in three communication steps, and the corresponding latency overhead for the consensus step, $D_{cn}$, is given in Eq. 5 of [26] and reproduced here:

2 $$\begin{aligned} D_{cn} = (4f+4)D_p + 3D_n \end{aligned}$$

The latency overhead of the traditional approach is about twice as the lightweight solution proposed in this article, as shown in Fig. 3a.

If the primary is faulty, a view change is needed to reach the consensus, which makes the latency overhead much higher. In our previous work, we have derived the total cost of latency in the presence of a successful round of view change, $D_{cc}$, which is given in Eq. 10 of [26] and reproduced here:

3 $$\begin{aligned} D_{cc} = fD_{fd}+(4f^2+7f+4)D_p+(2f+3)D_n \end{aligned}$$

The comparison of the total latency overhead in the presence of one round of successful view change for different replication degrees is shown in Fig. 3b (the fault detection time is assumed to be $200\,\mathrm{{ms}}$). Because the view change latency is proportional to $f^2$, the total latency overhead for the traditional approach increases nonlinearly with respect to the replication degree. Note that the log scale is used so that both latency curves are clearly shown for Fig. 3b.

Finally, because our mechanisms are based on the open-loop design mechanism proposed in [13], the latency overhead we report here is with respect to the baseline established in [13]. Note that because redundancy is not used in [13], it cannot tolerate faults in the supervisor component. Both our approach and the traditional Byzantine fault tolerance approach can tolerate crash and malicious faults in supervisor replicas. Because we use the same system model as that of the traditional approach, the hardware complexity (in terms of hardware redundancy) is identical for both approaches.

## Conclusion

In this article, we discussed the need of the strong dependability and trustworthiness for ICE, an emerging safety-critical real-time distributed system that is promising to revolutionize clinical operations for patient safety. By exploiting the fact that the supervisor logic is stateless, we proposed a set of lightweight mechanisms to enhance the dependability of ICEs. In our approach, the supervisor is replicated for fault and intrusion tolerance. Our mechanisms ensure that an ICE could continue operating correctly without stop even if a small portion of the replicas are faulty or have been compromised. Our cost analysis for the lightweight mechanisms shows that for the extra delays introduced by the mechanisms are not significant enough to impact the round-based normal operation of the ICEs while making the ICE significantly more resilient to hardware failures and cyber attacks.

We should also note that even though the context of our discussion in this article is integrated clinical environments, similar consensus requirement could arise in other biomedical applications [2, 9, 17, 22], such as teleoperation [14]. The approach we introduced in this article could be applied to solving these consensus problems in an efficient and robust manner.

## References

[1] ASTM F2761-2009 (2009) Medical devices and medical systems—essential safety requirements for equipment comprising the patient-centric integrated clinical environment (ICE), part 1: general requirements and conceptual model, ASTM International

[2] Arabnia HR, Tran QN (2016). Emerging trends in applications and infrastructures for computational biology, bioinformatics, and systems biology: systems and applications.

[3] Arney D, Pajic M, Goldman JM, Lee I, Mangharam R, Sokolsky O (2010) Toward patient safety in closed-loop medical device systems. In: Proceedings of the 1st ACM/IEEE International Conference on Cyber-Physical Systems. ACM, pp 139–148

[4] Castro M, Liskov B (2002). Practical byzantine fault tolerance and proactive recovery. ACM Trans Comput Syst.

[5] Chai H, Zhang H, Zhao W, Melliar-Smith PM, Moser LE (2013). Toward trustworthy coordination for web service business activities. IEEE Trans Serv Comput.

[6] Chai H, Zhao W (2014) Byzantine fault tolerance for services with commutative operations. In: 2014 IEEE International Conference on Services Computing (SCC). IEEE, pp 219–226

[7] Chai H, Zhao W (2014) Byzantine fault tolerant event stream processing for autonomic computing. In: 2014 IEEE 12th International Conference on Dependable, Autonomic and Secure Computing (DASC). IEEE, pp 109–114

[8] Chandra TD, Toueg S (1996). Unreliable failure detectors for reliable distributed systems. J ACM (JACM).

[9] Chen X, Li J, Hu L, Yang W, Lu L, Jin H, Wei Z, Yang JY, Arabnia HR, Liu JS (2016). The clinical significance of snail protein expression in gastric cancer: a meta-analysis. Hum Genom.

[10] Fischer MJ, Lynch NA, Paterson MS (1985). Impossibility of distributed consensus with one faulty process. J ACM (JACM).

[11] Foo Kune D, Venkatasubramanian K, Vasserman E, Lee I, Kim Y (2012) Toward a safe integrated clinical environment: a communication security perspective. In: Proceedings of the 2012 ACM Workshop on Medical Communication Systems. ACM, pp 7–12

[12] Kang W, Wu P, Sha L, Berlin RB Jr, Goldman JM (2012) Towards safe and effective integration of networked medical devices using organ-based semi-autonomous hierarchical control. University of Illinois, Technical Report

[13] Kim C, Sun M, Mohan S, Yun H, Sha L, Abdelzaher TF (2010) A framework for the safe interoperability of medical devices in the presence of network failures. In: Proceedings of the 1st ACM/IEEE International Conference on Cyber-Physical Systems. ACM, pp 149–158

[14] Lawrence DA (1993). Stability and transparency in bilateral teleoperation. IEEE Trans Robot Autom.

[15] Lee I, Sokolsky O (2010) Medical cyber physical systems. In: 2010 47th ACM/IEEE on Design Automation Conference (DAC). IEEE, pp 743–748

[16] Pajic M, Mangharam R, Sokolsky O, Arney D, Goldman JM, Lee I (2014) Model-driven safety analysis of closed-loop medical systems. IEEE Trans Ind Inform 10(1):3–16. doi:10.1109/TII.2012.2226594 10.1109/TII.2012.2226594 PMC3810414 24177176

[17] Shelton JO, Arabnia HR (2012) Brain imaging for diagnosis of schizophrenia: challenges, successes and a research road map. In: 2012 Ninth International Conference on Information Technology: New Generations (ITNG). IEEE, pp 578–583

[18] Taylor CR, Venkatasubramanian K, Shue CA (2014) Understanding the security of interoperable medical devices using attack graphs. In: Proceedings of the 3rd International Conference on High Confidence Networked Systems. ACM, pp 31–40

[19] Vasserman EY, Venkatasubramanian KK, Sokolsky O, Lee I (2012). Security and interoperable-medical-device systems, part 2: failures, consequences, and classification. IEEE Secur Priv.

[20] Venkatasubramanian KK, Vasserman EY, Sokolsky O, Lee I (2012). Security and interoperable medical device systems, part 1. IEEE Secur Priv.

[21] Wu PL, Kang W, Al-Nayeem A, Sha L, Berlin RB Jr, Goldman JM (2013) A low complexity coordination architecture for networked supervisory medical systems. In: Proceedings of the ACM/IEEE 4th International Conference on Cyber-Physical Systems. ACM, pp 89–98

[22] Yang W, Yoshigoe K, Qin X, Liu JS, Yang JY, Niemierko A, Deng Y, Liu Y, Dunker AK, Chen Z (2014). Identification of genes and pathways involved in kidney renal clear cell carcinoma. BMC Bioinform.

[23] Zhang H, Chai H, Zhao W, Melliar-Smith PM, Moser LE (2012). Trustworthy coordination for web service atomic transactions. IEEE Trans Parallel and Distrib Syst.

[24] Zhao W (2014) Application-aware byzantine fault tolerance. In: Proceedings of the 12th IEEE International Conference on Dependable, Autonomic and Secure Computing. IEEE, pp 45–50

[25] Zhao W (2014). Building dependable distributed systems.

[26] Zhao W (2015) Towards trustworthy integrated clinical environments. In: Proceedings of the IEEE 12th International Conference on Autonomic and Trusted Computing. IEEE, pp 452–459. doi:10.1109/UIC-ATC-ScalCom-CBDCom-IoP.2015.96

[27] Zhao W (2016). Performance optimization for state machine replication based on application semantics: a review. J Syst Softw.

[28] Zhao W, Babi M, Yang W, Luo X, Zhu Y, Yang J, Luo C, Yang M (2016) Byzantine fault tolerance for collaborative editing with commutative operations. In: 2016 IEEE International Conference on Electro Information Technology (EIT). IEEE, pp 0246–0251

[29] Zhao W, Melliar-Smith P, Moser LE (2012). Low latency fault tolerance system. Comput J.

[30] Zhao W, Yang W, Zhang H, Yang J, Luo X, Zhu Y, Yang M, Luo C (2016) High-throughput state-machine replication using software transactional memory. J Supercomput 72(11):4379–4398. doi:10.1007/s11227-016-1747-2 10.1007/s11227-016-1747-2 PMC5654484 29075049

